# Findings on Loneliness, Telehealth, and Advance Care Planning From the National Poll on Healthy Aging

**DOI:** 10.1093/geroni/igab046.671

**Published:** 2021-12-17

**Authors:** Erica Solway, Matthias Kirch, Dianne Singer, Jeffrey Kullgren, Cheryl Lampkin, Teresa Keenan, Alison Bryant, Preeti Malani

**Affiliations:** 1 University of Michigan, Ann Arbor, Michigan, United States; 2 AARP, Washington, District of Columbia, United States

## Abstract

The University of Michigan National Poll on Healthy Aging (NPHA) taps into the perspectives of older adults to inform health care policy and practice using a nationally representative sample of more than 2,000 adults age 50-80. Questions about lack of companionship and feelings of loneliness were tracked over three time points; 34% expressed feelings of loneliness in October 2018, 41% in June 2020, and 37% in January 2021. The NPHA also found that use of telehealth increased from 4% in May 2019 to 30% in June 2020 to 43% in January 2021. Finally, the NPHA found that 37% have completed both medical durable power of attorney and advance directive with 7% completing at least one of these documents in the first three months of the COVID-19 pandemic. These poll results can be used to inform actions by coalitions and organizations to advance state and federal policy.

